# Natural Compounds and Health Benefits of *Ganoderma capense*

**DOI:** 10.3390/molecules30102250

**Published:** 2025-05-21

**Authors:** Longshi Liu, Xinge Shi, Longkang Jia, Ran Wang, Chengwei Liu

**Affiliations:** 1 Key Laboratory for Enzyme and Enzyme-Like Material Engineering of Heilongjiang, College of Life Science, Northeast Forestry University, Harbin 150040, China; lls@nefu.edu.cn (L.L.); sxg2018@nedu.edu.cn (X.S.); a19819160271@outlook.com (L.J.); 2 College of Food and Biotechnology, Changchun Polytechnic University, Changchun 130033, China

**Keywords:** *Ganoderma capense*, medicinal fungi, bioactive compounds, pharmacological effects

## Abstract

*Ganoderma capense*, a member of the *Ganoderma* genus within the Polyporaceae family, has long been recognized for its high nutritional value and extensive use in traditional medicine. Its primary distribution is in China and South Africa, with the type locality being South Africa. This species is rich in a diverse array of bioactive compounds, including various polysaccharides, glycopeptide macromolecules, and various small-molecule compounds, such as sesquiterpenes, triterpenes, steroids, and alkaloids. Research indicates that these chemical constituents exhibit numerous pharmacological properties, including antioxidant, anti-inflammatory, and anti-tumor activities, as well as inhibition of acetylcholinesterase, reduction in blood lipids, and promotion of neural synapse growth. Apart from its use in traditional Chinese medicine, the components of *G. capense* are utilized globally for the treatment of a wide range of diseases, including Alzheimer’s disease, febrile convulsions, HIV, and diabetes. This underscores the extensive medical applications of *G. capense*, emphasizing its significance in contemporary and traditional healthcare. This review summarizes the latest research findings on the bioactive compounds and pharmacological effects of *G. capense*, compiled from databases such as PubMed, Web of Science, and Elsevier. This study aimed at providing researchers in this field with in-depth scientific insights and guidance, promoting further application and development in the pharmaceutical and food industries, and serving as a reference for subsequent exploration of active substances and the development of new disease treatments.

## 1. Introduction

*Ganoderma capense*, a fungus belonging to the Polyporaceae family, is widely known as the “medicinal mushroom” [1]. In traditional Chinese medicine, its mycelium, fruiting body, and spore powder can be used as medicine, and it has been regarded by generations of traditional Chinese medicine practitioners as a precious tonic that strengthens the body and nourishes the essence [2]. As an important medicinal fungus in the *Ganoderma* genus, *G. capense* has the effects of tonifying the kidneys, calming the nerves, boosting the immune system, and fighting inflammation and bacteria [3,4]. These effects are due to its rich content of various active metabolites, such as polysaccharides, triterpenoids, alkaloids, sterols, and other small-molecule compounds. Research conducted in the early stages of the study of *G. capense* focused on the composition of its compounds. For instance, in 1988, Yu et al. conducted a study in China on the chemical composition of *G. capense* mycelium following submerged fermentation, thereby ascertaining that it exerts certain therapeutic effects on diseases such as muscular dystrophy and atrophy [5]. Further research identified three new compounds: ganoderpurine and ganoine I and II [6]. Meanwhile, its pharmacological effects have also begun to attract widespread attention. For example, in 1980, Leng, W. et al. studied the pharmacological effects of *G. capense* fermentation broth and found that it has a certain hepatoprotective and detoxifying effect [7]. Because it is rich in nutrients and has significant medicinal value, it has the potential to be developed into health foods, medicines, etc. [8]. At present, preparations such as Boji injection and Boji tablets made from the fermentation culture products of the fungus have also been used in clinical practice to relieve symptoms such as muscle and joint pain, swelling, and skin edema [9].

This review article provides a comprehensive overview of the chemical structures and biological activities of the polysaccharide macromolecular compounds and small-molecule compounds, including terpenes and steroids, in *G. capense*. The primary objective of this review is to serve as a reference for subsequent pharmacological research and active compound mining of *G. capense.*

## 2. Polysaccharides

Polysaccharides represent a significant bioactive component of *G. capense*. Ten polysaccharides have been identified in *G. capense*. Wei, Z.H. et al. determined the content of compounds such as intracellular polysaccharides, hydrolyzed amino acids, and organic acids in *G. capense* and found that the polysaccharide content in *G. capense* was as high as 16% [10]. These polysaccharides are usually extracted with water and alkali solutions and are mainly composed of monosaccharides, such as glucose, arabinose, and xylose. These have been shown to possess activities including the treatment of malignant tumors and DPPH-hydroxyl radical activity, as demonstrated in the following experiments.

Initially, the liquid obtained by subjecting dried *G. capense* mycelial powder to soaking in petroleum ether for 24 h and subsequently refluxing it at 60 °C for 2 h in a round-bottomed flask was utilized as the experimental material for all the subsequent experiments. Li, N. et al. extracted *G. capense* mycelium by subjecting it to three cycles of boiling in water, each cycle lasting two hours. The mycelium was then deproteinized using a Sevag reagent, and the resulting mixture was further purified by DEAE Sepharose CL-6 and Sephadex G-75 column chromatography. This process resulted in the isolation of GCP50-1 [11]. It was identified that its main chain consisted of α-D-glucan, interspersed with (1→4)-α-D-glucopyranosyl groups, and the side chain is connected to the O-6 position with (1→4,6)-α-D-glucopyranosyl groups. GCPB-1b was also extracted from the mycelium of *G. capense* with an alkaline solution and purified using ion exchange and gel permeation column chromatography. The main chain is composed of (1→4)-linked α-D-glucopyranose residues and (1→4,6)-linked α-D-glucopyranose residues [12]. Huang Y. extracted a heteropolysaccharide from the mycelium using a sodium hydroxide alkaline solution and then purified it through a series of chromatographic columns (namely, DEAE-52 cellulose, DEAE Sepharose CL-6B, and Sephadex G-75) to yield a new polysaccharide, GCP50-2, whose main chain consists of (1→4)-linked β-D-xylose residues [13]. The structure of the substance is such that two branches are present at residue O-3, each of which constitutes a β-L-arabinopyranosyl residue terminated by a β-D-xylopyranosyl residue (1→3) and a β-D-xylopyranosyl residue terminated by a β-L-arabinopyranosyl residue (1→4). Yi, P. et al. extracted crude polysaccharides from the mycelia and then purified them by Sevag protein removal, anion exchange chromatography, and gel permeation chromatography to obtain polysaccharide GCPB-3, whose main chain is composed of β-L-arabinopyranosyluronic acid linked to β-D-xylose via 1→4 glycosidic bonds [14]. Huang, Y.T. extracted the fungal powder with deionized water (100 °C, 2 h), precipitated it with ethanol, and dialyzed it with deionized water. Finally, multiple polysaccharide components were obtained using DEAE Sepharose CL-6B ion exchange column chromatography and Sephadex G-75 gel columns and were named GCP50-2, GCP50-3, GCP70-1, GCP70-3, GCP 90-2, and GCP 90-3 based on the differences in the main chain and branch chain structures [4].

Furthermore, a plethora of glycopeptides have been identified within the mycelium of *G. capense*, exhibiting biological activities that encompass adjuvant therapy for tumors, liver protection, immunomodulation, and anti-tumor properties [15]. Lu, Y. et al. used methods such as oxidation with periodate and Smith degradation to confirm that the polysaccharide part of the glycopeptide is mostly composed of glucose, with two connection methods: 1→6 and 1→2 [16]. The glycopeptide was determined to be rich in various amino acids, such as glutamic acid, alanine, aspartic acid, and serine, using pre-column derivatization high-performance liquid chromatography. Lin, Y.Q. et al. used thin-layer chromatography and HPLC-ELSD to determine the monosaccharide composition of the glycopeptides, which was glucose: xylose: galactose: D-mannose = 1:0.0178:0.067:0.074 [17]. For the extraction part, extraction solution, relative molecular mass, and biological activity of *G. capense* polysaccharides, see Table 1.

## 3. Small Molecular Compounds

At present, 70 small-molecule compounds have been isolated from *G. capense*, including 13 sesquiterpenoids, 6 triterpenoids, 24 meroterpenoids, 17 steroids, and 10 other compounds. It has been demonstrated through experimental means that the aforementioned small molecules possess antioxidant, cytotoxic, and anti-cancer properties. In addition, they have been shown to exhibit anti-inflammatory, hypolipidemic, and neurotherapeutic effects, as well as therapeutic benefits in pemphigus vulgaris [16].

### 3.1. Terpenoid

Terpenoids are an important secondary metabolite in fungi [18]. They are usually classified according to the number of isoprene units in their molecular structure, such as monoterpenes with C10, sesquiterpenes with C15, diterpenes with C20, and triterpenes with C30. They are synthesized by the terpene synthase (TPS) acting on the polyisoprene precursor to form the basic backbone structure and then further modified by hydroxylation, dehydrogenation, acylation, or glycosylation to synthesize terpenoids with complex structures [19]. In addition, meroterpenoids are a distinctive class of compounds present in *Ganoderma*. Their structure, which is characterized by the presence of 1,2,4-trisubstituted phenyl groups and polyunsaturated terpene components, has been shown to have a variety of beneficial properties, such as antioxidant, immunosuppressive, and antiviral activities [20]. At present, several research teams have conducted in-depth research on the terpenoids in *G. capense*.

#### 3.1.1. Sesquiterpene

**Figure 1:**
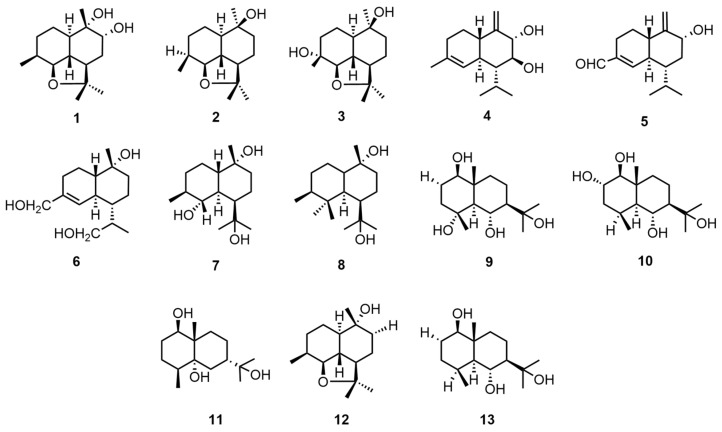
Structural formulas of compounds **1**–**13**.

Sesquiterpene compounds are widely found in nature, often in the form of oxygen-containing derivatives, such as alcohols and ketones [21]. Tan, Z. et al. diluted air-dried mycelium with 75% ethanol and then extracted the concentrated crude extract with water, followed by sequential extraction with petroleum ether (60–90 °C), ethyl acetate, and n-butanol. The extract was then purified using a series of petroleum ether–acetone gradients, petroleum ether–ethyl acetate gradient elution, and HPLC purification to obtain ganodermanol A–K (1–11), rel-(+)-(2aR,5R,5aR,8S,8aS,8bR)-decahydro-2,2,5,8-tetramethyl-2H-naphtho[1,8-bc] furan-5-ol (**12**), and eudesm-1β,6α,11-triol (**13**) [22]. The structures and absolute configurations of the compounds were identified through a combination of spectroscopic analysis, circular dichroism (CD), and Mo_2_(AcO)_4_-induced CD. The structural formulae of compounds **1**–**13** are shown in Figure 1. Please direct your attention to Table 2 in order to ascertain the biological activity and extraction sites of compounds **1**–**13**.

#### 3.1.2. Triterpenoid

Triterpenoids have diverse structures, including monocyclic, bicyclic, tricyclic, tetracyclic, and pentacyclic types, among which tetracyclic and pentacyclic triterpenoids are the most common. So far, more than 30,000 triterpenoid compounds have been identified [24]. Tan, Z. et al. concentrated the crude extract obtained after air-dried mycelium was extracted by refluxing with 75% ethanol and then sequentially extracted it with petroleum ether (60–90 °C), ethyl acetate, and n-butanol. Then, the extract was eluted through a petroleum ether–acetone and petroleum ether–ethyl acetate silica gel column and then purified by normal-phase semi-preparative HPLC using hexane–ethyl acetate methanol reversed-phase semi-preparative HPLC for further purification and separation to obtain four lanostane triterpenes: (24E)-15α-acetoxy-3-oxolanosta-8, 24-dien-26-oic acid (**14**), 15α-Acetoxy-3α-hydroxylanosta-8,24-dien-26-oic acid (**15**), (24E)-15α-acetoxy-3β-hydroxylanosta-8, 24-dien-26-oic acid (**16**), and 26-Methy-15α,22β-diacetoxy-7,9(11),24-trien-26-oic ester (**17**) [25]. The structures of these molecules were determined on the basis of extensive spectroscopic data analysis, including HRESIMS, 1D nuclear magnetic resonance (NMR), and 2D NMR.

**Figure 2:**
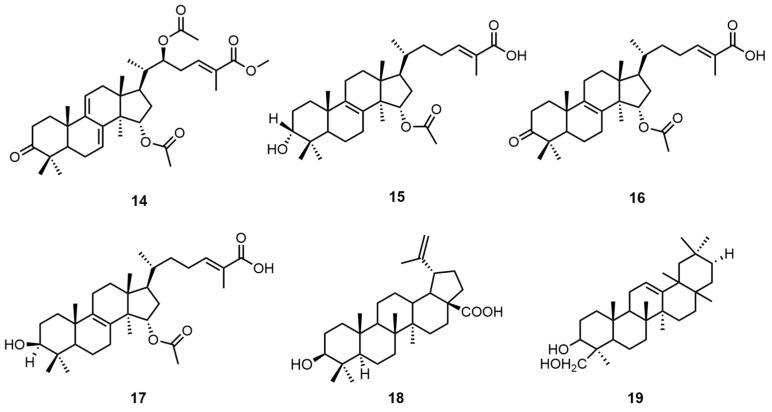
Structural formulas of compounds **14**–**19**.

Similarly, Peng, X.R. et al. used acetone to extract and concentrate the *G. capense* fruiting bodies and then treated them with macroporous adsorption resin and finally eluted them with a chloroform–acetone gradient to obtain the pentacyclic triterpene betulinic acid (**18**) [26]. Bean sapogenin-B (**19**) was extracted by similar means [6]. The structural formulas of triterpenoid compounds **14**–**19** are shown in Figure 2. Please direct your attention to Table 3 in order to ascertain the biological activity and extraction sites of compounds **14**–**19**.

#### 3.1.3. Meroterpenoid

Meroterpenoid compounds refer to those in the structure that are partially derived from the terpenoid biosynthesis pathway but also contain a non-terpene pathway source [27]. Liao, G.F. et al. used an 80% ethanol extract of mycelium in the ethyl acetate soluble fraction to sequentially perform MCI gel CHP 20 P, Sephadex LH-20, ODS (RP-18), and semi-preparative HPLC to obtain ganocapenoids A–D (**20**–**23**), ganoresain B (**24**), ganocalidin E (**25**), fornicin B (**26**), ganomycin I (**27**), zizhine A (**28**), amaurosubresin (**29**), co-chlearin I (**30**), ganocochlearin B (**31**), patchiene A (**32**), gano-mycin K (**33**), ganocalidin A (**34**), and spirolingzhine B (**35**) [28]. Then, the structures of the new compounds were determined through the application of spectroscopic methods, incorporating 1D and 2D NMR analyses, in addition to MS analyses.

**Figure 3:**
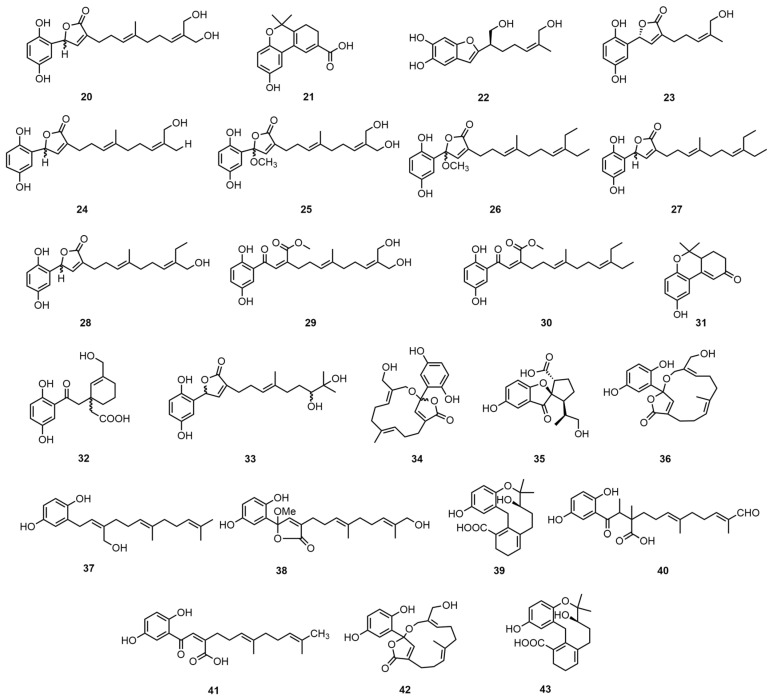
Structural formulas of compounds **20**–**43**.

Peng, X. et al. then used acetone to wash the chopped *G. capense* mycelium and then evaporated it under reduced pressure to obtain a crude extract. The extract was then passed through a methanol–water macroporous adsorption resin, eluted with a chloroform–methanol and chloroform–acetone silica gel column, and then was subjected to TLC to obtain ganocapensin A (**36**), ganomycin F (**37**), and fornicin E (**38**). Ganocapensin B (**39**), ganomycin E (**40**), and ganomycin C (**41**) were obtained by further using a reverse-phase C18 column and eluting the extract through a chloroform/methanol silica gel column, LH-20 (methanol) [29]. Peng, X.R. et al. extracted the *G. capense* fruiting bodies with acetone and concentrated them. They were first treated with a macroporous adsorption resin, followed by a normal-phase silica gel column sequentially using chloroform–methanol and chloroform–acetone to isolate cochlearin A (**42**). The compound was then treated with methanol–water from a 45% gradient to a 75% reverse-phase silica gel column and finally cut and gel-treated with a normal-phase silica gel column to obtain cochlearin B (**43**) [26]. The structures of the two meroterpenoids were characterized by 1DNMR, 2DNMR, mass spectrometry, and other spectroscopic techniques. The identification method was developed from earlier experiments of a similar nature [30]. The structural formulas of compounds **20**–**43** are shown in Figure 3. Please direct your attention to Table 4 in order to ascertain the biological activity and extraction sites of compounds **20**–**43**.

### 3.2. Steroid

Steroids are widely occurring natural compounds. Almost all living organisms can biosynthesize steroidal compounds themselves. Their molecular structures all contain a cyclopentane polyhydrophenanthrene carbon skeleton called a steryl nucleus [35]. Tan, Z. et al. extracted the mycelium of *G. capense* with petroleum ether (60–90 °C), eluted it with a gradient of petroleum ether–acetone and petroleum ether–ethyl acetate, and then separated and purified it by normal hexane–ethyl acetate normal phase semi-preparative HPLC and methanol reversed-phase semi-preparative HPLC to obtain demethylincisterol A3 (**44**) and 5α,9α-epidioxyergosta-6,8(18),22-triene-3β-ol (**45**) [25]. They also extracted the mycelium with ethyl acetate and petroleum ether–ethyl acetate silica gel column chromatography, followed by methanol Sephadex LH-20 column chromatography, methanol open MCI column chromatography, and reversed-phase semi-preparative HPLC to obtain 11α-hydroxy-21-hydroxy-demethylincisterol A3 (**46**) [25].

**Figure 4:**
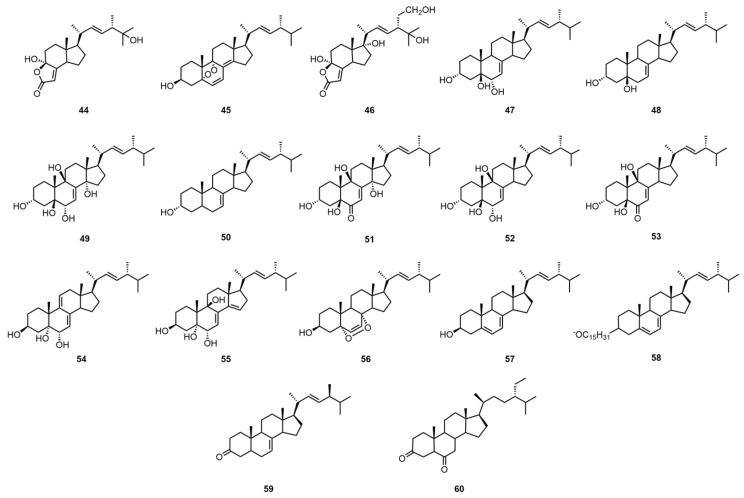
Structural formulas of compounds **44**–**60**.

Peng, X.R. et al. extracted the fruiting body with acetone, then eluted it with macroporous adsorption resin, followed by a normal-phase silica gel column fractionation and a gradient elution with chloroform–acetone. Then, it was purified by normal-phase HPLC (acetonitrile–water) with a chloroform–acetone system and P-TLC elution with chloroform–acetone to obtain (22E)-ergosta-7,22-dien-3β, 5α,6β-triol (**47**), (22E)-ergosta-7,22-dien-3β,5α-diol (**48**), ergosta-7,22-dien-3β,5α,6β, 5α,14α-pentol (**49**), (22E)-ergosta-7,22-dien-3β-ol (**50**), 3β,5α,9α,14β-tetrahydroxy-(22E)-ergosta-7,22-dien-6-one (**51**), (22E)-ergosta-7,22-dien-3β,5α,6β,9α-tetraol (**52**), (22E)-ergosta-7,9(11),22-dien-3β, 5α,6β-triol (**53**), (22E)-ergosta-7,14,22-dien-3β, 5α,6β-triol (**54**), 3β,5α,9α-trihydroxy-(22E)-ergosta-7,22-dien-6-one (**55**), and 5α,9α-epidioxy-(22E)-ergosta-6,22-dien-3β-ol (**56**) [26]. The identification of these compounds was facilitated by spectroscopic techniques, including 1DNMR, 2DNMR, and mass spectrometry. There are also articles mentioning that Yu, J.G. and others first extracted the fruiting body with ethanol and then with ether, and then eluted the extract with petroleum ether, ether, and acetone in sequence to obtain ergosterol (**57**), ergosterol palmitate (**58**), ergosterol-7,22-dien-3-one (**59**), and 5 α -sitostanedione-3,6 (**60**) [6]. The team then identified these compounds by high-resolution mass spectrometry (HRMS) with infrared spectroscopy (IR). The structural formulas of compounds **44**–**60** are shown in Figure 4. For information pertaining to the biological activity and extraction sites of compounds **44**–**60**, please refer to Table 5.

### 3.3. Others

**Figure 5:**
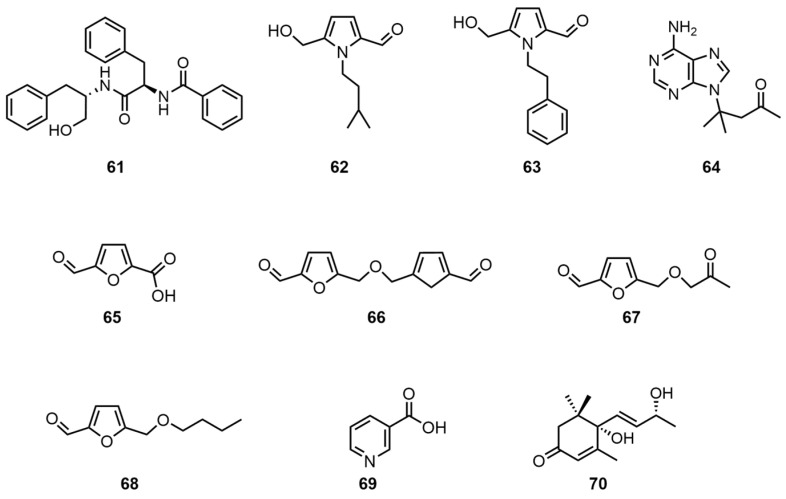
Structural formulas of compounds **61**–**70**.

Small-molecule compounds, such as amides, ganoderic alkaloids, and furan derivatives, were also isolated from the thin-skinned *G. capense* fruiting bodies. Peng, X.R. et al. utilized microporous adsorption resin for the extraction of the fruiting bodies, followed by normal-phase silica gel column fractionation. A chloroform–acetone gradient elution and acetonitrile–water HPLC were then employed to obtain aurantiamide (**61**) [26]. Yu, J.G. et al. used 95% ethanol to extract the mycelium and then successively extracted with ether, chromatographed with methanol–petroleum ether on a silica gel column, and eluted with a gradient of petroleum ether–acetone to obtain ganoine I (**62**) by silica gel column chromatography [30]. Alternatively, the extracted liquid was acetylated and then successively subjected to silica gel thin-layer separation, petroleum ether–ethyl acetate development, and petroleum ether–dichloromethane recrystallization to obtain a white crystalline ganoine II (**63**). In addition, Yu, J.G. et al. treated mycelium with cationic resin, alkalized it with hydroxylamine, and eluted it with ethanol, then performed multiple silica gel column chromatography and finally recrystallized it with acetone to obtain ganoderpurine (**64**) [30]. In addition, articles have been published that report on the use of 95% ethanol reflux for the extraction of mycelium by Yu, J.G. and colleagues. Successive extraction methods employed included ether, dichloromethane–methanol, hexane–ethyl acetate elution, and vacuum distillation. The final product obtained was 5-hydroxymethyl furfuraldehyde (**65**). The process of elution was concentrated, and the resulting solid was allowed to stand in order to obtain the dehydrated disulfide 1,1′-di-α-glyoxal dimethyl ether (**66**), 5-acetyloxymethyl furfuraldehyde (**67**), or 5-butoxymethyl furfuraldehyde (**68**). These were obtained by thin-layer preparation using a silica gel column and hexane–ethyl acetate. Finally, nicotinic acid (**69**) was obtained through a series of chemical processes, including ethanol extraction, treatment with cationic resin, alkalization with ammonia, heating, and elution with ac-acetone. The product was then subjected to multiple silica gel column chromatography steps, followed by recrystallization [6]. Tan, Z. et al. extracted an ethyl acetate extract (8.9 g) with a petroleum ether–ethyl acetate gradient and then sequentially eluted with methanol. Sephadex LH-20 chromatography column fractionation and methanol reversed-phase semi-preparative HPLC were used to obtain bluemenol A (**70**) [22]. The structural formulas of compounds **61**–**70** are shown in Figure 5. For information pertaining to the biological activity and extraction sites of compounds **61**–**70**, please refer to Table 6.

## 4. Pharmacological Effect

### 4.1. Antioxidant

Reactive oxygen species (ROS) have a strong damaging effect on DNA and proteins, and this damage can lead to aging and the development of many other important diseases, such as cancer, cardiovascular disease, and neurodegeneration [46]. Terpenoids have the potential to act as antioxidants by scavenging ROS in cells [47].

The majority of studies on scavenging effects against oxidants have been conducted in vitro, as Peng, X. et al. found when they added heterocyclic compounds **26**, **27**, **37**, **38**, **39**, **40**, and **41** to a DPPH radical methanol solution. The half-inhibitory concentrations (IC_50_) values exhibited a range from 6.00 ± 0.11 to 8.20 ± 0.30 µg/mL, and it was found that these had a stronger DPPH radical scavenging effect than the positive control (Trolox antioxidant, IC_50_ value of 3.63 ± 0.17 μg/mL) [29]. Yan, C. et al. dissolved the polysaccharides in distilled water at concentrations of 0.125 to 25 mg/mL, added 0.5 mL 0.1% H_2_O_2_, and used DPPH free radical and hydroxyl radical activity assays with ascorbic acid as a positive control. It was found that the polysaccharides in the mycelium all had concentration-dependent scavenging abilities [48]. For example, the antioxidant capacity of GCPB-1b was measured using this DPPH assay, and the effective concentration (EC_50_) value for the inhibition of DPPH radicals was 3.23 μM, which removed 60.2% of the DPPH radicals [12]. Huang, Y. et al. and Yi, P. et al. used the same method to determine that GCP50-2 and GCPB-3 had DPPH radical scavenging rates of 10.9% and 15.0%, respectively [13]. In addition, polysaccharides can also improve the cell proliferation index, alter the expression levels of genes related to skin cell aging, and have an anti-epidermal aging effect, so they can be used to make anti-aging creams and other cosmetics [49].

### 4.2. Cytotoxicity and Anti-Cancer Effect

Cytotoxic anti-tumor drugs are currently one of the main treatments for malignant tumors. They are a type of chemotherapy drug that can directly kill tumor cells or inhibit tumor cell growth and proliferation [50]. Research in this area has achieved certain results. For example, Tan, Z. et al. extracted sesquiterpene compound **3** from the mycelium, which was cytotoxic to the human liver cancer cell line HCT 116. In vitro experiments were performed, with paclitaxel and leflunomide used as positive controls. The IC_50_ values for HCT116, NCI-H1650, BGC823, Daoy, and HepG2 cells were found to be 0.000809, 0.945, 0.00177, 0.00933, and 0.0249 µM, respectively. Cell viability was measured by the conversion of 3-(4,5-dimethylthiazol-2-yl)-2,5-diphenyltetrazolium bromide (MTT) to purple formazan crystals. The IC_50_ of the antagonists were 24.5 and 12.2 μM. Sesquiterpene compound **4** is cytotoxic to human colon cancer cells HCT 116, human gastric adenocarcinoma cells BGC 823, human medulloblastoma cells Daoy, and human liver cancer cells HepG2, with IC_50_ values of 16.6, 49.9, 31.1, 27.9, and 37.9 μM, respectively. The IC_50_ values of sesquiterpene compound **6** for the human cancer cell lines NCI-H1650 and Daoy were 28.9 and 35.5 μM, respectively [22]. Later, they used the same method to measure triterpene compound **14**, which showed moderate cytotoxic activity against NCI-H1650, with an IC_50_ value of 22.3 μM, and the cytotoxic activity of steroidal compound **44** against the human cancer cell line HCT 116, with an IC_50_ value of 17.4 μM [25]. Huang, Y.T. also used the MTT method to measure the same polysaccharide GCP70-3, which has a strong inhibitory effect on the proliferation of human gastric cancer cells SGC-7901, human cervical cancer cells Hela, and human nasopharyngeal cancer cells CNE-1. At a concentration of 3200 μg/mL, the inhibition rates were 50.44%, 41.69%, and 52.30%, respectively [4].

Song, A.R. et al. measured the toxicity of sesquiterpene compound **13** on canine renal epithelial cells MDCK. After treating the monolayer of MDCK cells with compound **13** for 48 h, the OD492 value was measured in a microplate reader, and the cytotoxic concentration 50% (CC_50_) was determined to be 85.39 μM. The MDCK cells were then infected with TCID 50, which is related to the pathogenicity of the virus, and zanamivir was designated as the positive control, and its EC_50_ value was determined to be 15 μM. The virus inhibition rate was determined to calculate the 50% effective concentration. The EC_50_ of compound **13** was 0.14 nM, indicating that it has extremely strong anti-influenza virus activity [23]. Tran, P.T. et al. used bone marrow cells that were isolated from the femurs and tibias of male mice and then treated them with heterocyclic compound **27**, which was found to inhibit the phosphorylation of the extracellular signal-regulated kinase signaling pathway (ERK), c-Jun N-terminal kinase (JNK), and mitogen-activated protein kinase p38 (p38 MAPK), induced by the receptor activator of nuclear factor-κB ligand (RANKL) pathway, and the expression of cellular oncogene fos (c-Fos) and nuclear factor of activated T-cells, cytoplasmic, calcineurin-dependent 1 (NFATc 1) transcription factors, thereby inhibiting osteoclast formation [51]. This mechanism can be used in anti-rheumatic drugs. Zhou, J. et al. mentioned that compound **70** is an anti-tumor compound with anti-proliferative effects. It showed an effective dose 50% (ED_50_) of 20 μg/mL in an in vitro test of quinone reductase inducers in cultured Hepa1c1c7 mouse liver cancer cells [43].

Kitagawa, K. et al. studied the anti-tumor and anti-epithelial–mesenchymal transition inhibitory effects of heterocyclic compound **33** through an in vivo xenograft study, in which mice were inoculated with bladder cancer cells, and the compound was administered intratumorally. They found that it significantly inhibited the non-muscle-invasive KK 47 tumor cells (*p* < 0.01) and muscle-invasive T24 tumor cells (*p* < 0.01). Even when the compound was used at a high dose (1.0 mg/individual) intra-tumorally, it not only inhibited tumor growth in KK 47 and T24 cells (*p* = 0.009 and *p* = 0.003, respectively) but also did so without significant adverse events [34].

### 4.3. Anti-Inflammatory

Inflammation is an adaptive response of the immune system to harmful stimuli. Inflammation can be caused by infections, pathogen invasion, cell damage, excessive autoimmunity, and toxic compounds. It is a complex and important physiological and pathological response [52]. The main physical manifestations of inflammation are redness, swelling, heat, pain, and loss of function in the affected area. Inflammation has a significant impact on human health, so research on inflammation is currently very important [53]. Zhou, Y. et al. stimulated macrophages with Baozi polysaccharide peptide complexes and found that it can regulate the production of NO and the normal expression of inducible nitric oxide synthase (iNOS) through the nuclear factor kappa-light-chain-enhancer of the activated B cells (NFκ-B) signaling pathway and the phosphorylation and degradation of inhibitor of NF-κB (IκB), thus demonstrating its inflammatory regulatory effect on macrophages [54].

Peng, X.R. discovered amide compound **61** from the fruiting body, and then He, R.B. et al. used the CCK 8 technique to evaluate cell viability in vitro and found that the compound restored the viability of human proximal tubular epithelial cells in a dose-dependent manner at concentrations of 25, 50, and 100 μM. In subsequent in vivo experiments, by ligating the ceca of mice with a puncture needle, then suturing them to induce sepsis, and administering the compound orally, it was found that it could improve renal tubular damage and inflammation induced by ischemia and the cecal ligation and puncture by acting on the gastrin-releasing peptide receptor [38].

### 4.4. Lower Blood Sugar and Lipids

Diseases caused by high blood sugar levels have always been a great challenge for mankind. As posited by Yu, J.G., a furan derivative, designated 65, was identified in mycelium. Zhang, X. and Shimizu, A. utilized this compound on Balb/c 3T3 A31 clone 19 cells, which demonstrated sensitivity to insulin or insulin-like growth factor stimulation. This resulted in a glucose absorption rate of 50.68%, thereby confirming that both the compound and its derivatives possess hypoglycemic activity [40,41]. In addition, Liao, G.F. et al. assessed the effect of heterocyclic compound **34** on lipid accumulation in HepG2 cells by oil red O staining. When it reached 20 μM, it could reduce liver lipid accumulation by increasing cholesterol efflux and reducing cholesterol synthesis [28].

Wang et al. subsequently demonstrated in vitro that heterocyclic compound **27** exhibited a superior dual inhibitory effect on α-glucosidase and HMG-CoA reductase in comparison to the positive control atorvastatin. Furthermore, it was observed that **27** effectively reduced blood glucose and lipid levels. In comparison, the medication was found to be comparable to rosiglitazone, with a reduction in blood glucose levels of 15 mM on the first day and maintenance of this level subsequently. In vivo experiments using an insulin-resistant male mouse model demonstrated that the liver glycogen concentration of the experimental group was significantly higher than that of the untreated control group (*p* < 0.01). These findings suggest that this compound may improve insulin resistance and serve as a potential therapeutic agent for diabetes [55].

### 4.5. Neurotherapeutic Effect

Etukudo, E. M.’s paper systematically discusses that sesquiterpenoids **22**, **25**, **29**, and **30** all have acetylcholinesterase (Ache) inhibitory effects, which can repair neuronal degeneration and treat Alzheimer’s disease associated with decreased Ache levels, as well as treat myasthenia gravis [56,57]. Liao, G.F. et al. also found that heterocyclic compound **30** has an effective Ache inhibitory effect, with an IC_50_ value of 8.2 μM, and when the rate of neural differentiation was examined after 72 h of action at 10 μM, it was found to promote moderate neurite growth [28].

Zheng, F.X. et al. conducted an in vivo experiment by inducing a mouse model of thermal convulsions and found that the incidence of severe convulsions in the experimental group was lower than that in the saline control group after intraperitoneal injection of *G. capense*. The content of cytokines in the hippocampal homogenates was then determined by ELISA; it was found that *G. capense* can reduce excitotoxic brain damage caused by interleukin-1 beta (IL-1β) and reduce the expression level of the apoptosis protein caspase-3 in the hippocampus of rats, thereby reducing the incidence of epilepsy in rats with recurrent febrile seizures [58]. In addition, Wang, Z.Y. observed family members with hereditary cerebellar ataxia who were injected intramuscularly with *G. capense* mycelium extract and found that their symptoms were relieved [59].

### 4.6. Antibacterial Activity

Given the prevalence of antibiotic-resistant microorganisms, there is an urgent need for the development of novel antibiotics. *G. capense* contains substances with corresponding functions.

Vazirian, M. dissolved compounds **50** and **59** in chloroform at concentrations of 100–3.125 mg/mL and 10–1.25 mg/mL, respectively, and then inoculated them into culture media containing Staphylococcus aureus, Bacillus subtilis, Escherichia coli, Pseudomonas aeruginosa, and Candida albicans. The compounds (**50** and **59**) were subjected to an incubation process at a temperature of 37 °C for a duration of 24 h. Thereafter, the average zone diameter (in millimeters) of the inhibition zone was measured at a concentration of 10 milligrams per milliliter. The minimum concentration at which an inhibition zone was observed was defined as the MIC. The minimum inhibitory concentration (MIC) values for compound **50** were determined to be 5, 5, >10, >10, and 5 milligrams per milliliter (mg/mL), suggesting the absence of an inhibition zone in both Escherichia coli and Pseudomonas aeruginosa. A similar pattern was observed for compound **59**, with MIC values of 5, 5, >10, >10, and 5 mg/mL, consistent with the aforementioned findings. However, when the concentration of compound **50** was 10 mg/mL, the inhibition zone diameters of Staphylococcus aureus, Bacillus subtilis, and Candida albicans were 9.7 ± 0.3, 9.6 ± 0.7, and 9.6 ± 0.9, respectively. The mean values ± standard deviation for the inhibition zone diameters of compounds **59** were 9.7 ± 0.6, 9.5 ± 0.5, and 9.6 ± 0.1 (millimeters ± standard deviation), respectively [37].

The antibacterial effect may be attributable to two distinct mechanisms: the inhibitory effect of active oxygen and the induction of intracellular protein leakage in bacterial cells [60]. To date, a paucity of studies has been published on the antimicrobial activity of *G. capense*. Moreover, the majority of these studies have been conducted in vitro, and the mechanisms underlying the antimicrobial and antiviral activities of *G. capense* remain to be elucidated. Despite the high concentration of bioactive compounds (carbohydrates, glycosides, triterpenoids, phenolic compounds, and tannins) present in the extracts, these compounds primarily demonstrate antimicrobial activity when present in a mixed form [61].

### 4.7. Other Pharmacological Activities

Stuart, K.L. and Mogana, R. et al. found that compound **70** is a compound with activity comparable to that of abscisic acid, has Ache activity, and exhibits moderate anti-leishmanial activity [44,45]. Ngai, P.H. isolated lectins from mycelium extracts that have strong mitogenic activity compared to cowpea globulin A and strong antiproliferative activity against leukemia (L1210 and M1) cells and hepatoma (HepG 2) cells [62]. Tan, Z. et al. and Hapuarachchi, K. all detected the concentration of viral capsid protein through in vitro antigen capture enzyme-linked immunosorbent assays and found that sesquiterpenoid compound **4**, triterpenoid compounds **14**, **15**, **16**, heterocyclic compound **27**, and sterol compound **45** had anti-HIV capabilities, and the IC_50_ values were determined to be 37.9, 23.5, 46.7, 21.6, 30.1, and 46.7 μM, respectively [22,25,31]. In other respects, Liu, Y.M. et al. mentioned that Professor Xuan Guowei often uses Phellinus linteus when treating alopecia areata because it can enhance macrophage activation and thus secrete interleukin, inhibit the proliferation of T lymphocytes and B lymphocytes, and thus promote hair growth [63].

**Figure 6:**
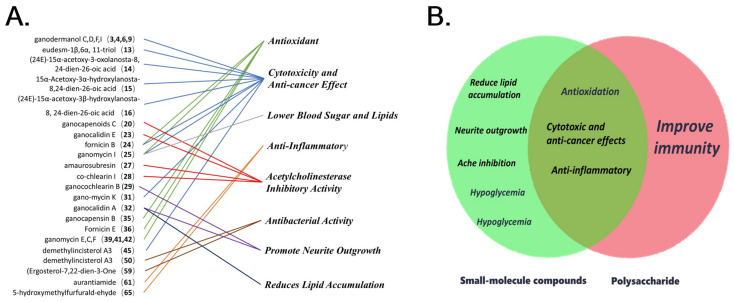
This chart depicts the pharmacological value of the main compounds (**A**). This Venn diagram depicts the connection and relationship between the direct pharmacological value of polysaccharides and small-molecule compounds (**B**).

Then, Li, W. used the 2HRZE/4HR regimen on patients with AIDS and secondary tuberculosis and found that the regimen had an effective rate of 91.67% after the addition of low-molecular-weight ganderma glycopeptide, which was higher than the control group’s 77.08% (*p* < 0.05), confirming that the addition of low-molecular-weight ganoderic acid peptide can effectively boost immunity [64]. Zeng, L.G. used *G. capense* and acyclovir to treat recurrent genital herpes, and the cure rate reached 81%, which was a significant improvement compared to the 47% cure rate of the control group that took levamisole tablets [65]. For a visual representation of the pharmacological activities corresponding to each small molecule compound, as well as the relationship between small molecule compounds and polysaccharides, please refer to Figure 6.

## 5. Conclusions and Prospection

In summary, the polysaccharides and small-molecule chemical constituents in *G. capense* show high medicinal value. *G. capense* has been widely used to treat various diseases, including alopecia, diabetes [56], and areata [63]. Nevertheless, research on its chemical constituents remains comparatively restricted, with the majority of studies concentrating on terpenes, steroids, and other components. It is evident that the mining and exploration of other active ingredients remains inadequate. Therefore, it is necessary to conduct more in-depth and extensive research on the medicinal value of *G. capense*. Although the anti-tumor activity of *G. capense* polysaccharides has been preliminarily explored, its specific mechanism of action and application prospects still require further research in order to fully tap its potential and apply it clinically.

In addition, the triterpenoids in *G. capense* have a wide range of applications in medicine. To increase the production of triterpenoids, genetic engineering can be used to overexpress key genes in the terpene synthesis pathway, such as HMGR (3-hydroxy-3-methylglutaryl-coenzyme A reductase), SQS (squalene synthase), and LS (lanosterol synthase), and the transcription factor [66], which negatively regulates terpene synthesis, is simultaneously inhibited, thereby promoting the synthesis of triterpenoids. With the development of genome sequencing technology and the gradual improvement of the analysis of natural product biosynthesis pathways, the functional identification of biosynthesis genes for the key active ingredients of this fungus and metabolic pathway reconstruction can be carried out in the future. By transferring these genes to a host, such as Saccharomyces cerevisiae, an efficient heterologous biosynthesis platform can be established that can enable the large-scale production of important components of this fungus, thereby reducing costs and improving the controllability and stability of the product. In addition, synthetic biology methods can further optimize yield and quality, providing a new approach for the clinical drug development of *G. capense*.

This review aims to provide basic data and strong evidence for future pharmacological mechanism research on this fungus by organizing and summarizing the medicinal activities of the polysaccharides and small-molecule compounds of *G. capense*. This will not only deepen our understanding of its pharmacological effects but also open up new directions for its clinical application in anti-tumor, anti-inflammatory, and immune regulation. By further optimizing its biosynthesis pathway and exploring effective drug application schemes, the application prospects of *G. capense* in modern medicine will be even broader.

## Figures and Tables

**Table 1:** Polysaccharide components and bioactivity in *G. capense*.

Solution	Name	N_W_	Polysaccharide	Monosaccharide Composition	Biological Activities	Reference
Hot water	GCP50-1 GCP50-2 GCP50-3 GCP70-1	1.5 × 10^4^ Da 3167 Da 8123 Da 10,685 Da	glucan glucan	glucose glucose	—— —— —— ——	[11]
	GCP70-3	32,436 Da	heteropolysaccharide	D-mannose glucose galactose xylose rabinose	Inhibitory activity against tumor cells MGC-803, etc.	[4]
	GCP90-2 GCP90-3	6381 Da 5808 Da	heteropolysaccharide	D-mannose glucose galactose rhamnose xylose arabinose	—— ——	
Sodium-hydroxide	GCPB-2	1.03 × 10^5^ Da	heteropolysaccharide	xylose arabinose	DPPH· hydroxyl radical activity	[13]
	GCPB-1b	2847 Da	glucan	glucose	Inhibits the proliferation of MGC-803 cells	[12]
	GCPB-3	1.24 × 10^5^ Da	heteropolysaccharide	xylose arabinose	DPPH· hydroxyl radical activity	[14]

**Table 2:** Biological activity of small-molecule compounds **1**–**13**.

Number	Chemical Compound	Biological Activities	Materials	References
**1**	ganodermanol A	-	mycelium	[22]
**2**	ganodermanol B	-	mycelium	[22]
**3**	ganodermanol C	Cytotoxic to human liver cancer cell line HCT 116	mycelium	[22]
**4**	ganodermanol D	Cytotoxic and anti-HIV activities against human liver cancer cell lines HCT116, BGC823, Daoy and HepG_2	mycelium	[22]
**5**	ganodermanol E	-	mycelium	[22]
**6**	ganodermanol F	Toxic to human cancer cells NCI-H1650 and Daoy	mycelium	[22]
**7**	ganodermanol J	-	mycelium	[22]
**8**	ganodermanol H	-	mycelium	[22]
**9**	ganodermanol I	Cytotoxic to human liver cancer cell line HCT116	mycelium	[22]
**10**	ganodermanol G	-	mycelium	[22]
**11**	ganodermanol K	-	mycelium	[22]
**12**	rel-(+)-(2aR,5R,5aR,8S,8aS,8bR)-decahydro-2,2,5,8-tetramethyl-2H-naphtho[1,8-bc]furan-5-ol	-	mycelium	[22]
**13**	eudesm-1β,6α,11-triol	Ability to inhibit influenza virus	mycelium	[22,23]

**Table 3:** Biological activity of small-molecule compounds **14**–**19**.

Number	Chemical Compound	Biological Activities	Materials	References
**14**	(24E)-15α-acetoxy-3-oxolanosta-8,24-dien-26-oicacid	Weak anti-HIV activity	mycelium	[25]
**15**	15α-Acetoxy-3α-hydroxylanosta-8,24-dien-26-oicacid	Weak anti-HIV activity	mycelium	[25]
**16**	(24E)-15α-acetoxy-3β-hydroxylanosta-8,24-dien-26-oicacid	Weak anti-HIV activity	mycelium	[25]
**17**	26-Methy-15α,22β-diacetoxy-7,9(11),24-trien-26-oicester	Cytotoxic activity against the human cancer cell line NCI-H1650	mycelium	[25]
**18**	betulinic acid	-	fruit bodies	[26]
**19**	bean sapogenin-B	-	mycelium	[6]

**Table 4:** Biological activity of small-molecule compounds **20**–**43**.

Number	Chemical Compound	Biological Activities	Materials	References
**20**	ganocapenoids A	-	Fruit bodies	[28]
**21**	ganocapenoids B	-	Fruit bodies	[28]
**22**	ganocapenoids C	AchE inhibitory activity	Fruit bodies	[28]
**23**	ganocapenoids D	Neurotrophic factor	Fruit bodies	[28]
**24**	ganoresain B	-	Fruit bodies	[28]
**25**	ganocalidin E	AchE inhibitory activity	Fruit bodies	[28]
**26**	fornicin B	Cytotoxic to Hep-2 cells, antioxidant activity	Fruit bodies	[28,29,31]
**27**	ganomycin I	Anti-diabetic effect, anti-HIV protease, antioxidant activity, anti-rheumatic	Fruit bodies	[28,29,31,32]
**28**	zizhine A	-	Fruit bodies	[28]
**29**	amaurosubresin	AchE inhibitory activity	Fruit bodies	[28,33]
**30**	co-chlearin I	Neurotrophic factor AchE inhibitory activity	Fruit bodies	[28]
**31**	ganocochlearin B	-	Fruit bodies	[28]
**32**	patchiene A	AchE inhibitory activity	Fruit bodies	[28]
**33**	gano-mycin K	Inhibits epithelial–mesenchymal transition	Fruit bodies	[28,34]
**34**	ganocalidin A	Results in a decrease in lipid accumulation in HepG2 cells	Fruit bodies	[28]
**35**	spirolingzhine B	Promotes the proliferation of neural stem cells	Fruit bodies	[28,32]
**36**	ganocapensin A	Antioxidant activity	Fruit bodies	[29]
**37**	ganomycin F	Antioxidant activity	Fruit bodies	[29]
**38**	fornicin E	Antioxidant activity	Fruit bodies	[29]
**39**	ganocapensin B	Antioxidant activity	Fruit bodies	[29]
**40**	ganomycin E	Antioxidant activity	Fruit bodies	[29]
**41**	ganomycin C	Antioxidant activity	Fruit bodies	[29]
**42**	cochlearin A	-	Fruit bodies	[26]
**43**	cochlearin B	-	Fruit bodies	[26]

**Table 5:** Biological activity of small-molecule compounds **44**–**60**.

Number	Chemical Compound	Biological Activities	Materials	References
**44**	demethylincisterol A3	Cytotoxic to the human cancer cell line HCT 116	mycelium	[25]
**45**	5α,9α-epidioxyergosta-6,8(14),22-triene-3β-ol	Weak anti-HIV activity	mycelium	[25]
**46**	11α-hydroxy-21-hydroxy-demethylincisterol A3	-	mycelium	[25]
**47**	(22E)-ergosta-7,22-dien-3β, 5α,6β-triol	-	fruit bodies	[26]
**48**	(22E)-ergosta-7,22-dien-3β,5α-diol	-	fruit bodies	[26]
**49**	ergosta-7,22-dien-3β,5α,6β, 5α,14α—pentol	-	fruit bodies	[26]
**50**	(22E)-ergosta-7,22-dien-3β-ol	Antibacterial activity	fruit bodies	[27,36,37]
**51**	3β,5α,9α,14β-tetrahydroxy-(22E)-ergosta-7,22-dien-6-one	-	fruit bodies	[26]
**52**	(22E)-ergosta-7,22-dien-3β,5α,6β,9α-tetraol	-	fruit bodies	[26]
**53**	(22E)-ergosta7,9(11),22-dien-3β, 5α,6β-triol	-	fruit bodies	[27]
**54**	(22E)-ergosta-7,14,22-dien-3β, 5α,6β-triol	-	fruit bodies	[26]
**55**	3β,5α,9α-trihydroxy-(22E)-ergosta-7,22-dien-6-one	-	fruit bodies	[26]
**56**	5α,9α-epidioxy-(22E)-ergosta-6,22-dien-3β-ol	-	fruit bodies	[26]
**57**	ergosterol	Antineoplastic, antiviral, immunomodulatory, and antibacterial	mycelium	[6]
**58**	ergosterol palmitate	-	mycelium	[6]
**59**	ergosterol-7,22-dien-3-One	Antibacterial activity	mycelium	[6,37]
**60**	5α-sitostanedione-3,6	-	mycelium	[6]

**Table 6:** Biological activity of small-molecule compounds **61**–**70**.

Number	Chemical Compound	Biological Activities	Materials	References
**61**	Aurantiamide	Anti-tubular inflammation	fruit bodies	[27,38]
**62**	Ganoine I	Reduces cholesterol and blood lipids, protects the liver, and fights inflammation	mycelium	[30,39]
**63**	Ganoine II	Reduces cholesterol and blood lipids, protects the liver, and fights inflammation	mycelium	[30,39]
**64**	Ganoderpurine	-	mycelium	[30]
**65**	5-Hydroxymethylfurfural	Hypoglycemic activity and inhibition of platelet aggregation	mycelium	[6,40,41]
**66**	1,1′di-a-furaldehydic dimethyl ether	-	mycelium	[6]
**67**	5-acetoxymethyl-furfuraldehyde	-	mycelium	[6]
**68**	5-butoxymethyl-furfuraldehyde	-	mycelium	[6]
**69**	Pyridine-3-carboxylic acid	Promotes cell metabolism and dilates blood vessels	mycelium	[6,42]
**70**	Bluemenol A	Acetylcholinesterase activity and anti-leishmanial activity	mycelium	[22,43,44,45]

## Data Availability

No new data were created or analyzed in this study. Data sharing is not applicable to this article.
